# Downregulation of ACE2 expression by SARS-CoV-2 worsens the prognosis of KIRC and KIRP patients via metabolism and immunoregulation

**DOI:** 10.7150/ijbs.57802

**Published:** 2021-05-10

**Authors:** Qian Tang, Yue Wang, Ling Ou, Jieling Li, Kai Zheng, Hui Zhan, Jiayu Gu, Guibao Zhou, Shouxia Xie, Jianping Zhang, Wei Huang, Shaoxiang Wang, Xiao Wang

**Affiliations:** 1 School of Pharmacy, Jinan University, Guangzhou 510630, China.; 2 School of Pharmaceutical Sciences, Shenzhen University Health Science Center, Shenzhen 518000, China.; 3 Department of Pharmacy, Shenzhen People's Hospital (The Second Clinical Medical College, Jinan University; The First Affiliated Hospital, Southern University of Science and Technology), Shenzhen 518020, China.; 4 Bacteriology & Antibacterial Resistance Surveillance Laboratory, Shenzhen Institute of Respiratory Diseases, Shenzhen People's Hospital (The Second Clinical Medical College, Jinan University; The First Affiliated Hospital, Southern University of Science and Technology), Shenzhen 518020, China.

**Keywords:** ACE2, TMPRSS2, renal cancer, SARS-CoV-2, overall survival

## Abstract

**Background:** Angiotensin-converting enzyme 2 (ACE2) and transmembrane serine protease 2 (TMPRSS2) allow entry of severe acute respiratory syndrome coronavirus 2 (SARS-CoV-2) into host cells and play essential roles in cancer therapy. However, the functions of ACE2 and TMPRSS2 in kidney cancer remain unclear, especially as kidneys are targets for SARS-CoV-2 infection.

**Methods:** UCSC Xena project, the Cancer Genome Atlas (TCGA), and Gene Expression Omnibus (GEO) databases (GSE30589 and GSE59185) were searched for gene expression in human tissues, gene expression data, and clinical information. Several bioinformatics methods were utilized to analyze the correlation between ACE2 and TMPRSS2 with respect to the prognosis of kidney renal clear cell carcinoma (KIRC) and kidney renal papillary cell carcinoma (KIRP).

**Results:** ACE2 expression was significantly upregulated in tumor tissue, while its downregulation was associated with low survival in KIRC and KIRP patients. TMPRSS2 was downregulated in KIRC and KIRP, and its expression was not correlated with patient survival. According to clinical risk factor-based prediction models, ACE2 exhibits predictive accuracy for kidney cancer prognosis and is correlated with metabolism and immune infiltration. In an animal model, ACE2 expression was remarkably downregulated in SARS-CoV-2-infected cells compared to in the control.

**Conclusion:** ACE2 expression is highly correlated with various metabolic pathways and is involved in immune infiltration.it plays a crucial role than TMPRSS2 in diagnosing and prognosis of kidney cancer patients. The overlap in ACE2 expression between kidney cancer and SARS-CoV-2 infection suggests that patients with KIRC or KIRP are at high risk of developing serious symptoms.

## Introduction

The coronavirus disease 2019 (COVID-19) pandemic has caused a worldwide healthcare emergency, with more than 70,000,000 infected patients and 1,600,000 deaths since December 2019 (https://coronavirus.jhu.edu/map.html). Considerable efforts have been made to explore the pathogenesis of the disease and develop effective therapeutic strategies for severe acute respiratory syndrome coronavirus 2 (SARS-CoV-2). However, the complicated molecular mechanisms underlying the attacks on the human body by SARS-CoV-2 remain unclear.

Angiotensin-converting enzyme 2 (ACE2) and transmembrane serine protease 2 (TMPRSS2) facilitate host cell entry for SARS-CoV-2. SARS-CoV-2 employs ACE2 as an entry receptor into host cells through the plasma membrane, or it can gain entry into host cells by endocytosis via releasing a protease 1, 2 that engages TMPRSS2 for S protein priming 1. Both of these strategies enable the infection and pathogenesis of COVID-19 3, 4. Recent reports have shown that ACE2 5 and TMPRSS2 6 are highly expressed in the kidneys. In addition to the lungs, the human kidney is a target for SARS-CoV-2 infection 7. Epidemiological evidence has also revealed that many COVID-19 patients display kidney injury 8, 9, suggesting that patients with kidney disease have a high risk of SARS-CoV-2 infection. The distribution and expression levels of ACE2 and TMPRSS2 may play a role in the development of kidney disease. Typically, ACE2 participates in the physiological and pathological regulation of the renin-angiotensin system (RAS) 10, which regulates homeostasis by reducing blood pressure, and exerting anti-inflammatory and anti-proliferation effects 11. TMPRSS2 functions as a host factor for some viruses 12, 13 and involves signal transduction between cancer cells and the extracellular environment 13. Hence, the changes in *ACE2* and *TMPRSS2* messenger RNA (mRNA) expression caused by cancer or SARS-CoV-2 might compromise its protective role and accelerate disease progression.

ACE2 is a key negative modulator of RAS and is crucial for maintaining homeostasis 11, 14. RAS is an essential physiological and pathological modulator in numerous organs, such as the heart, lungs, and kidneys 10. The activation of a recently identified RAS axis, known as the angiotensin-converting enzyme 2/angiotensin-(1-7)/mitochondrial assembly receptor (ACE2/Ang 1-7/MasR) axis, is a critical part of the gastric mucosa, pulmonary systems, and cancer 15. It acts as a negative modulator of angiotensin-2 (Ang II) activity, which induces tumor progression in intrahepatic cholangiocarcinoma 16, 17. Ang-(1-7) is an endogenic heptapeptide hormone that moderates the biological activity of Mas, and is a part of the axis formed by ACE2. This biological activity is dysregulated in some cancers 18, 19. Moreover, low ACE2 expression may be a useful indicator of poor prognosis in hepatocellular carcinoma 20. While increased activity of ACE2 has been observed in healthy individuals, low ACE2 activity is usually related to the occurrence of cancer 21. Similarly, *TMPRSS2* is repeatedly altered in primary prostate cancer and is a crucial factor facilitating SARS-CoV-2 infection 6. Tripathi et al. revealed that TMPRSS2 levels are reduced in renal carcinoma compared to in normal renal tissue 22. Bao et al. reported that ACE2 and TMPRSS2 are less abundant in tumors than in healthy controls 23.

Renal cell carcinoma (RCC) is a common malignant tumor that originates from the urinary tubule epithelial system of the renal parenchyma 14, and is the sixth most frequently diagnosed disease in men and the tenth in women, with >140,000 RCC-related deaths occurring every year 15. RCC is classified into various specific tumors with different molecular, histological, and genetic characteristics 16. Among these, kidney renal clear cell carcinoma (KIRC) and kidney renal papillary cell carcinoma (KIRP) are the two most predominant histological subtypes 17, accounting for 85%-95% of RCC cases. In addition, the incidence of RCC has noticeable regional differences, and the morbidity in European and American countries is significantly higher than in Asian countries 18. Cancer has been identified as an independent risk factor for SARS-CoV-2 infection 19, and cancer patients have a higher risk of contracting COVID-19, developing complications, and experiencing deteriorating health 20. Although some studies have explored the function of ACE2 in pan-cancer 21 and clear cell RCC (ccRCC) 22, studies on ACE2 and TMPRSS2 in kidney cancer remain scarce. Therefore, it is essential to investigate the function of ACE2 and TMPRSS2 in RCC, and their potential prognostic impact on patients with renal cancer after COVID-19 infection.

In the present study, we aimed to explore the expression and prognostic power of ACE2 and TMPRSS2 in KIRC and KIRP, as well as the changes in ACE2 and TMPRSS2 in cells and an animal model with SARS-CoV infection, using a bioinformatics approach. In addition, the potential post-infection risks in KIRC and KIRP patients were evaluated to provide a reference for clinical treatment.

## Materials and methods

### Data collection

Genes expressed in human tissue were downloaded from the Genotype-Tissue Expression (GTEx) RNA-Seq gene expression profiling datasets for 31 human tissue types, compiled from 7845 human samples from the UCSC Xena project (https://xenabrowser.net/datapages/). The gene expression data and clinical information of patients were extracted from the Cancer Genome Atlas (TCGA) database (https://portal.gdc.cancer.gov/). Among a total of 611 RNA-seq cases and 526 KIRC cases, 46.6% patients were <60 years-old and 53.4% were ≥60 years-old, 34.7% were female, and 65.2% were male (Supplementary Table 1). Among 321 RNA-seq cases and 285 KIRP cases, 41.4% were <60 years-old and 57.9% were >60 years-old, 26.7% were female, and 73.3% were male (Supplementary Table 2). The microarray data of GSE30589 and GSE59185 were obtained from the Gene Expression Omnibus (GEO) database (https://www.ncbi.nlm.nih.gov/geo/). We selected genome sequencing data from 12 SARS-CoV-infected Vero E6 cell samples and nine control samples, and three mouse lung tissue samples from mice infected with SARS-CoV and three normal mouse lung tissue samples.

### Differential expression analysis

The gene expression profiles of ACE2 and TMPRSS2 were obtained from five datasets, including 72 normal and 539 tumor samples with KIRC from TCGA, 32 normal and 289 tumor samples with KIRP from TCGA, 28 normal kidney samples from the GTEx, and the microarray data of GSE30589 and GSE59185 from the GEO. The empirical analysis of digital gene expression data was performed using the edgeR package 23 in R (v3.6.3) to identify the differentially expressed genes in KIRC and KIRP. Similarly, the linear models for microarray data (limma) R package 24 were used to assess the differential expression of ACE2 between SARS-CoV-infected samples and control samples in GSE30589 and GSE59185. The difference in the gene expression levels between normal and tumor tissues was analyzed using the exact test in edgeR and the empirical Bayes statistical test in limma. Subsequently, the *P*-value, false discovery rate (FDR), and fold change (logFC) were derived. Genes with |logFC| ≥0.5, and *P*<0.05, or FDR<0.05 were identified as differentially expressed genes.

### Protein expression data and mechanism analysis

The immunohistochemical data of ACE2 and TMPRSS2 protein expression in kidney cancer and normal tissue were obtained from the Human Protein Atlas (HPA) (https://www.proteinatlas.org/). Immunostaining photographs were quantified using a computerized image analysis system (Image-Pro Plus 6.0; Media Cybernetics, Silver Spring, MD, USA)^25^. Staining area and cumulative optical density were measured to calculate the mean optical density. The DNA methylation levels of ACE2 and TMPRSS2 in KIRC and KIRP were obtained from UALCAN (http://ualcan.path.uab.edu/). Correlation analysis between ACE2 and copy number variation (CNV) was performed using Gene Set Cancer Analysis (GDSC) (https://www.cancerrxgene.org/). An FDR <0.05 was considered statistically significant.

### Survival analysis and prediction models

Survival curves were plotted using the Kaplan-Meier method and compared using the *P*-value package in R. The risk scores of patients were calculated based on multivariate Cox regression using the survival package in R, using several candidate factors and the overall survival to predict patient survival. The survival receiver operating characteristic (ROC) package was used to plot ROC curves and calculate the area under the curve (AUC) values at 3 years in R, according to the risk scores. AUC values between 0.9-1 are indicate excellent predictive power, AUC values between 0.8-0.9 are good, AUC values between 0.7-0.8 are fair, AUC values between 0.6-0.7 are poor, and AUC values between 0.5-0.6 indicate a lack of predictive power 26, 27. Typically, the accuracy of the predictive model is optimal when the AUC is >0.6. Survival curves were plotted by dividing the patients into high- and low-risk groups based on the median risk score of survival. The distribution of the risk score and patient survival status were also visualized to assess the prognostic differences between the two groups. The nomograms for individual predictions were generated to forecast the 3- and 5-year overall survival of patients according to the risk score based on ACE2 expression and clinical risk factors using R software. Differences between groups were considered statistically significant at *p* <0.05.

### Gene set enrichment analysis

Gene set enrichment analysis (GSEA) was used to explore the statistical correlation between phenotypic class distinction and predefined gene sets, using the gene expression profiles of tumor samples in GSEA v4.0.3. The samples were divided into high- and low-risk groups based on their ACE2 transcript levels. Kyoto Encyclopedia of Genes and Genomes (KEGG) gene sets (v7.1) and hallmark gene sets (v7.1) (http://software.broadinstitute.org/gsea/msigdb/collections.jsp) were used as references. The normalized enrichment score (NES) reflected the degree to which a gene set was overrepresented in the groups, and the gene sets with *P*<0.05, and NES>1 were considered significant.

### Co-expression network analysis

Weighted gene co-expression network analysis (WGCNA) was used to assess the co-expression of genes with *ACE2* in KIRC and KIRP in R, and the resulting network was visualized using Cytoscape v3.7.2. Pearson' s correlation coefficient was used to identify the correlation between ACE2, TMPRSS2 and selected hub genes using Statistical Package for the Social Sciences (SPSS) v21.

### Drug sensitivity analysis

A total of 265 small molecules or drugs were obtained from the Genomics of Drug Sensitivity in Cancer database (GDSC: https://www.cancerrxgene.org/). Spearman's coefficient was used to analyze the correlation between gene expression and drug sensitivity.

### Statistical analysis

The hazard ratios (HRs) and *P*-value were calculated to determine the correlation between the clinicopathological features and patients' overall survival using SPSS v21 by univariate and multivariate Cox regression analysis, based on TCGA datasets. The Kruskal-Wallis test was used to examine the correlation between gene expression and clinical characteristics. Kaplan-Meier survival curves were compared using the log-rank test. The correlation between ACE2 levels and immune cell markers was evaluated using Pearson's correlation. Differences were considered to be statistically significant at *p* <0.05.

## Results

### ACE2 and TMPRSS2 are widely expressed in various human tissues

**Figure 1:**
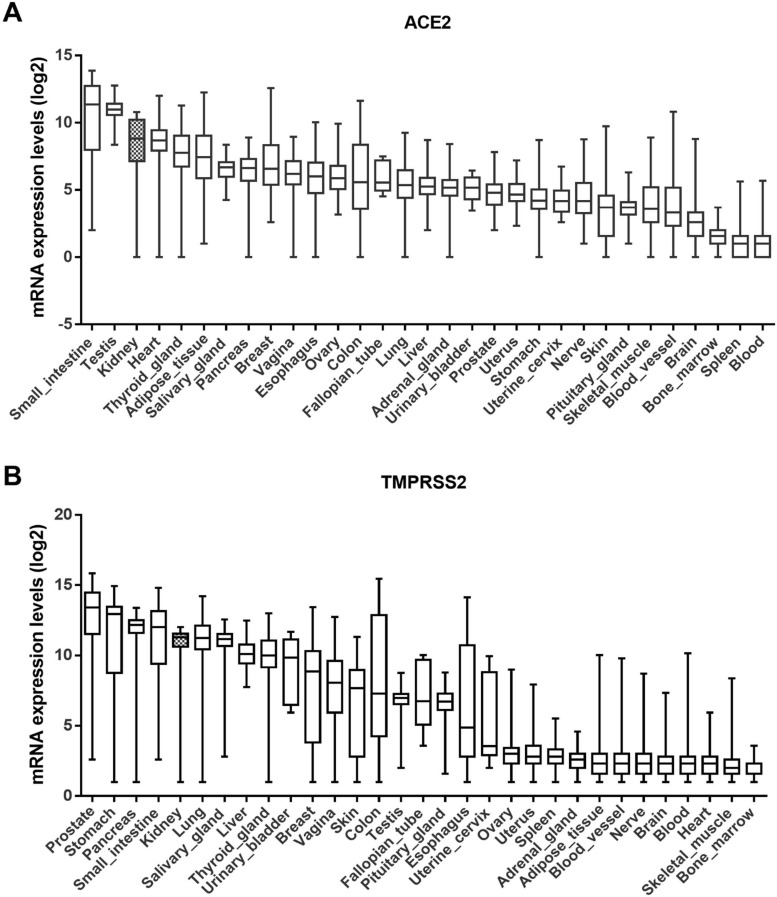
** Expression levels of ACE2 and TMPRSS2 in various tissues.** (A-B) ACE2 and TMPRSS2 have been expressed widely in various human tissues from the GTEx RNA-seq gene expression profiling datasets. ACE2, angiotensin-converting enzyme 2; TMPRSS2, transmembrane serine protease 2; GTEx, genotype-tissue expression.

The GTEx dataset includes 31 tissue types compiled from 7845 human samples. According to this dataset, ACE2 is highly expressed in the small intestine, testis, kidney, and heart tissue, and moderately expressed in the pancreas, breast, esophagus, colon, and lungs (Fig. 1A). Similarly, the expression level of TMPRSS2 was higher in the kidneys than in the lungs (Fig. 1B). Although their expression in the lungs is linked to disease, including SARS-CoV-2, ACE2 and TMPRSS2 are widely expressed throughout the human body.

### Differential expression of ACE2 and TMPRSS2 in KIRC and KIRP

**Figure 2:**
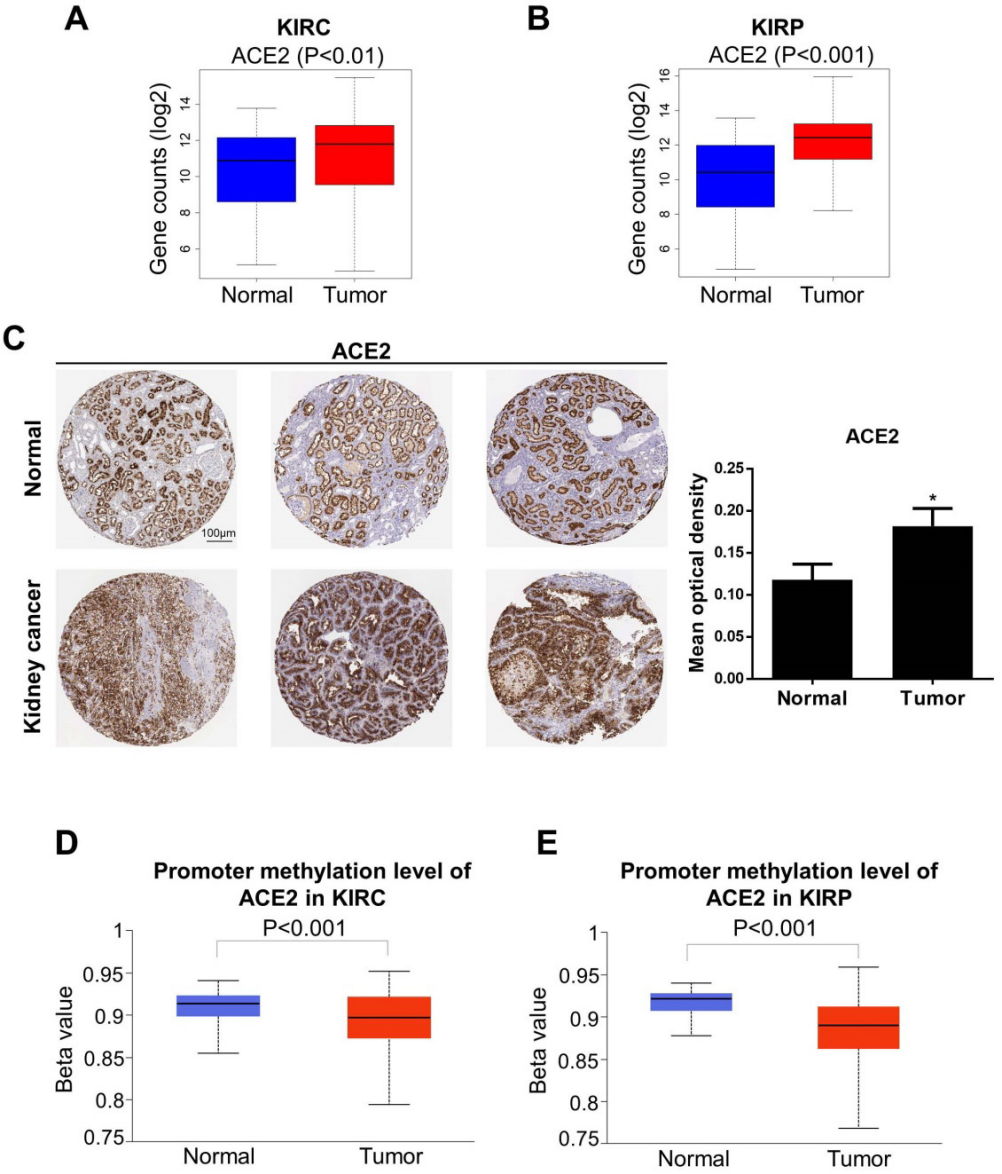
** Altered ACE2 expression levels in kidney cancer.** (A-B) ACE2 expression was increased in KIRC and KIRP based on the combination of GTEx and TCGA datasets. (C) The protein expression of ACE2 in kidney cancer from the HPA. (D-E) KIRC and KIRP presented a decrease in DNA methylation levels of ACE2. These data were obtained by UALCAN. ACE2, angiotensin-converting enzyme 2; KIRC, kidney renal clear cell carcinoma; KIRP, kidney renal papillary cell carcinoma; GTEx, genotype-tissue expression; TCGA, the cancer genome atlas; HPA, human protein atlas.

**Figure 3:**
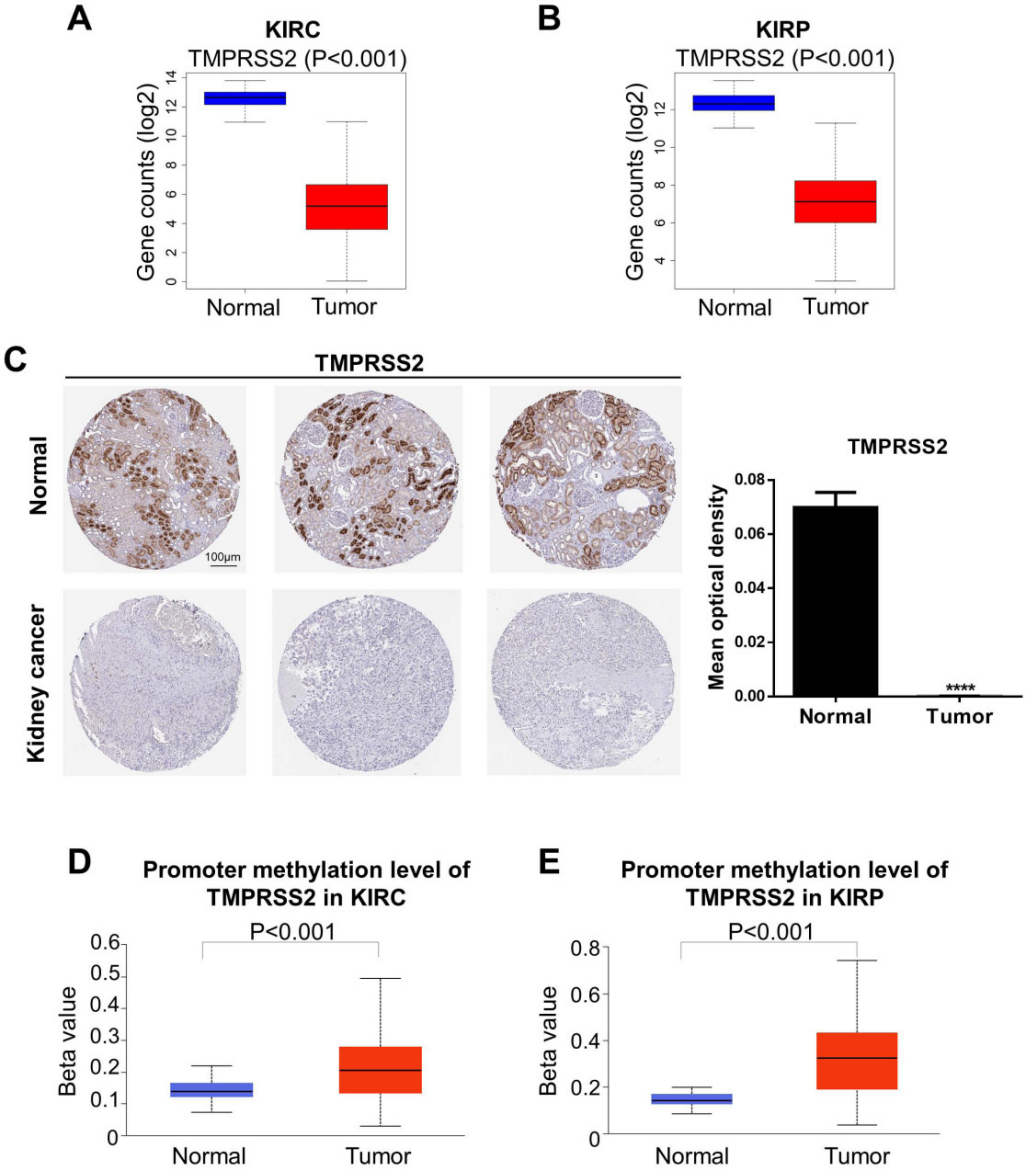
** Change of TMPRSS2 expression levels in kidney cancer.** (A-B) TMPRSS2 expression was decreased in KIRC and KIRP based on the combination of GTEx and TCGA datasets. (C) The protein expression of TMPRSS2 in kidney cancer from the HPA. (D-E) KIRC and KIRP presented an increase in DNA methylation levels of TMPRSS2. These data were obtained by UALCAN. TMPRSS2, transmembrane serine protease 2; KIRC, kidney renal clear cell carcinoma; KIRP, kidney renal papillary cell carcinoma; GTEx, genotype-tissue expression; TCGA, the cancer genome atlas; HPA, human protein atlas.

We next analyzed the levels of ACE2 and TMPRSS2 in normal and tumor tissue by combining the GTEx and TCGA datasets (KIRC: 100 normal and 539 tumor samples; KIRP: 60 normal and 289 tumor samples). To further explore the changes in the protein and DNA methylation levels of ACE2 and TMPRSS2 in KIRC and KIRP, we accessed the HPA to obtain immunohistochemical images and the UALCAN database to verify the methylation level. We found that ACE2 expression was upregulated significantly in tumor tissues in KIRC and KIRP (Fig. 2A-B). The protein expression level of ACE2 was higher in tumor tissue than in normal tissue (Fig. 2C). Moreover, the DNA methylation levels of the ACE2 promoter in KIRC and KIRP were significantly downregulated in tumor tissue compared to in normal tissue (Fig. 2D-E), whereas TMPRSS2 expression was low in both KIRC and KIRP (Fig. 3A-B). In addition, TMPRSS2 protein levels were lower in tumor tissue compared to in the control tissue (Fig. 3C), while DNA methylation levels were dramatically higher in tumor samples for KIRC and KIRP (Fig. 3D-E). However, the expression of ACE2 and TMPRSS2 was almost unaffected by CNV (Supplementary Figure 1).

We also evaluated the correlation between these proteins and clinical risk factors using the Kruskal-Wallis test based on TCGA datasets. In KIRC, a high level of ACE2 was observed in females and in the early TNM stage. In KIRP, the expression levels of both ACE2 and TMPRSS2 were higher in the early stages than in advanced TNM stages (Supplementary Figure 2). These data suggest that ACE2 is an appropriate marker for the early diagnosis of KIRC or KIRP.

### Clinicopathological characteristics and survival analysis of kidney cancer

The correlation between clinicopathological factors and overall survival in KIRC and KIRP patients was analyzed using univariate and multivariate Cox regression analyses. Poor overall survival was significantly associated with age, T stage, TNM stage, tumor grade, and ACE2 expression in KIRC patients, and was also related to T stage, TNM stage, and ACE2 expression in KIRP patients. Multivariate analysis indicated that both TNM stage and ACE2 expression were independent risk factors for OS in KIRC. The results are presented in Supplementary Tables 1 and 2.

**Figure 4:**
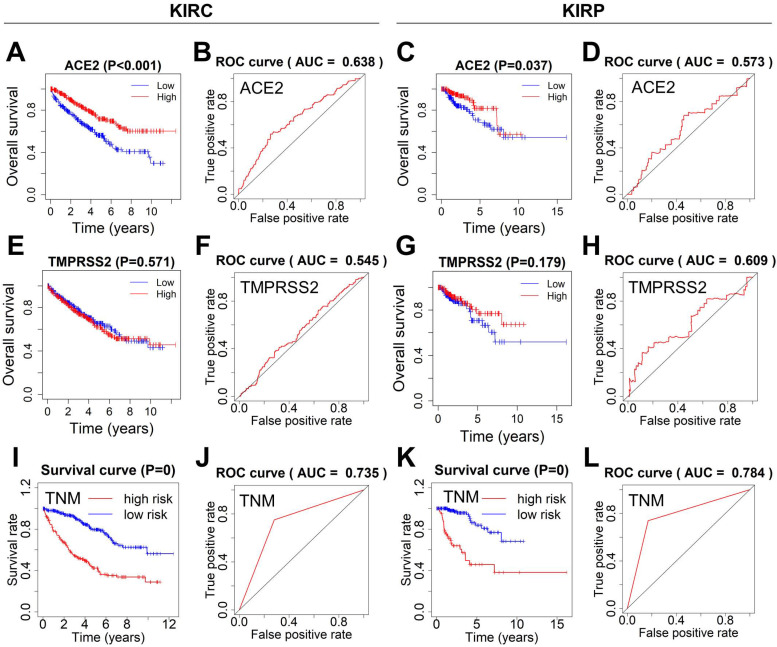
** Survival analysis and prognosis accuracy of different factors.** (A-D) Kaplan-Meier survival curves and ROC curves of ACE2 in KIRC and KIRP patients. (E-H) Kaplan-Meier survival curves and ROC curves of TMPRSS2 in KIRC and KIRP patients. (I-L) ROC and survival curves of the models according to TNM staging. ROC, receiver operating characteristic; ACE2, angiotensin-converting enzyme 2; KIRC, kidney renal clear cell carcinoma; KIRP, kidney renal papillary cell carcinoma; TMPRSS2, transmembrane serine protease 2; TNM, tumor-node-metastasis.

Next, the survival curves of ACE2 and TMPRSS2 were plotted using the Kaplan-Meier method, and ROC curves were used to estimate the power of ACE2, TMPRSS2, and TNM for predicting survival. KIRC or KIRP patients with lower ACE2 expression showed poor overall survival. The ROC curves of ACE2 indicated ordinary predictive power in KIRC (AUC=0.638), but not in KIRP (AUC=0.573) (Fig. 4A-D). In contrast, TMPRSS2 expression was not significantly associated with overall survival, and TMPRSS2 had no predictive power in either KIRC or KIRP (Fig. 4E-H). The TNM stage model in both KIRC and KIRP revealed an optimal predictive performance in high-risk groups that correlated significantly with poor survival (Fig. 4I-L).

### Predictive models of kidney cancer based on ACE2 and clinical risk factors

**Figure 5:**
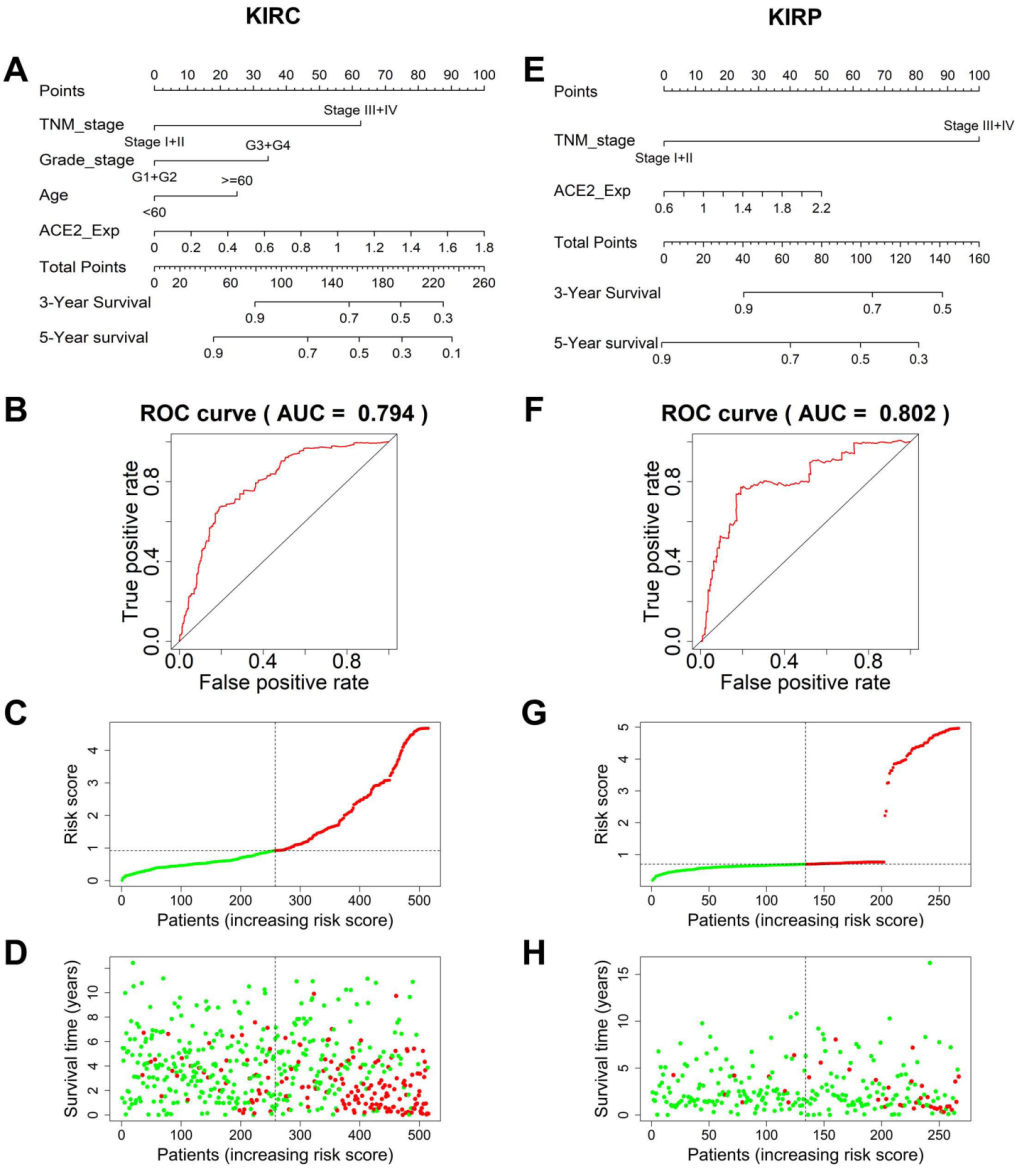
** Prediction models to forecast the prognosis of KIRC and KIRP patients.** (A) The nomogram integrating TNM stage, tumor grade, age, and ACE2-based risk score for 3-year survival rate prediction in KIRC and KIRP (E). (B) The ROC curves of the nomogram for 3-year survival rate prediction in KIRC and KIRP (F). (C, G) The patients' risk score distribution in ascending order was divided into low-risk (green) and high-risk (red) groups. (D, H) The plot of patients' survival time and status increases risk scores, and the red and green dots represent dead and alive, respectively. TNM, tumor-node-metastasis; ACE2, angiotensin-converting enzyme 2; KIRC, kidney renal clear cell carcinoma; ROC, receiver operating characteristic; KIRP, kidney renal papillary cell carcinoma.

To establish an accurate predictive model, we integrated ACE2 and overall survival-related factors to construct a nomogram. The ROC curve showed that the ACE2-TNM-grade-age-integrated nomogram in KIRC had a better predictive ability than models based on an ACE2 or TNM stage alone (AUC=0.794, Fig. 5A-B). The patients' risk scores were ranked in ascending order and divided into high- and low-risk groups based on the median point. Patients with high-risk scores had high mortality rates and short survival (Fig. 5C-D). Similarly, the predictive model based on combining ACE2 and TNM stage was more accurate in predicting KIRP outcomes (AUC=0.802, Fig. 5E-F), and patients with high-risk scores also had high mortality rates and short survival (Fig. 5G-H).

### Pathway analysis and co-expression network analysis of ACE2

**Figure 6:**
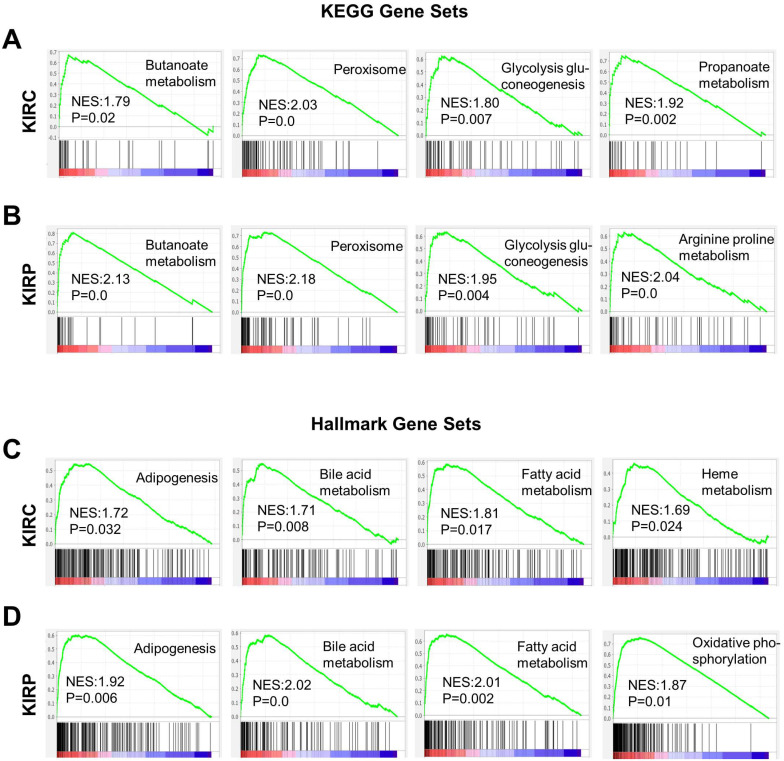
** Gene sets enrichment analysis based on ACE2 expression in KIRC and KIRP.** (A-B) Representative enriched gene sets according to KEGG in KIRC and KIRP, respectively. (C-D) Representative enriched gene sets according to hallmark gene clusters in KIRC and KIRP, respectively. KEGG, Kyoto encyclopedia of genes and genomes; ACE2, angiotensin-converting enzyme 2; KIRC, kidney renal clear cell carcinoma; KIRP, kidney renal papillary cell carcinoma.

Based on the correlation between ACE2 and the prognosis of renal cancer, we analyzed the potential mechanisms by which ACE2 could influence KIRC or KIRP. Gene set enrichment analysis was conducted based on the ACE2 expression profiles in tumor samples. Gene sets were closely correlated with metabolic pathways in both KIRC and KIRP, most of which were upregulated in the high-risk group (Fig. 6A-D). These enriched gene sets include butanoate metabolism, glycolysis gluconeogenesis, peroxisome, propanoate metabolism, arginine, and proline metabolism based on the KEGG database. According to hallmark gene sets, the high-risk group was significantly associated with bile acid metabolism, fatty acid metabolism, adipogenesis, heme metabolism, oxidative phosphorylation, and peroxisomes.

**Figure 7:**
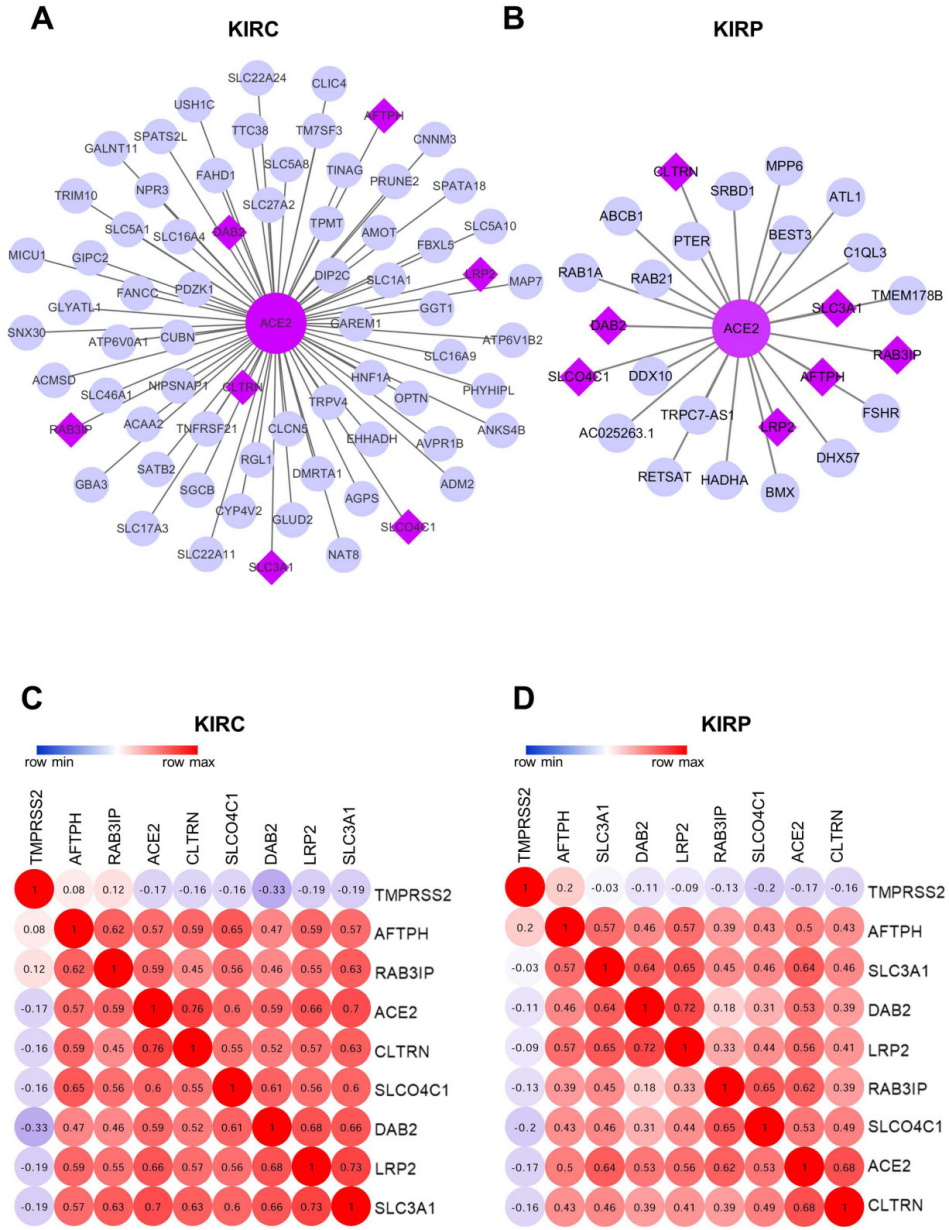
** Co-expression network analysis.** (A-B) The co-expression network of ACE2 in KIRC and KIRP, respectively. The big purple circle is ACE2. The small grey circles are the co-expressed genes, and the purple diamonds are the mutual co-expressed genes between KIRC and KIRP. (C-D) The correlation between ACE2, TMPRSS2 and 7-mutual co-expressed genes in KIRC and KIRP, respectively. ACE2, angiotensin-converting enzyme 2; TMPRSS2, transmembrane serine protease 2; KIRC, kidney renal clear cell carcinoma; KIRP, kidney renal papillary cell carcinoma.

The WGCNA package was employed to investigate the genes that were co-expressed with ACE2 in KIRC and KIRP. The co-expressed genes with a correlation >0.5 were extracted and visualized using Cytoscape (Fig. 7A-B). Consequently, seven mutually co-expressed genes were identified, overlapping between KIRC and KIRP, and all were implicated in the modulation of metabolism and membrane transport, and were positively correlated with ACE2 expression, but only weakly negatively correlated with TMPRSS2 expression. At the same time, ACE2 and TMPRSS2 were weakly negatively correlated (Fig. 7C-D). We next analyzed the correlation between these genes and overall survival. In line with our findings for ACE2, the low expression of seven mutually co-expressed genes were correlated with poor survival in KIRC, and the remaining four genes (*RAB3IP*, *SLC3A1*, *AFTPH*, and *CLTRN*) were associated with patient survival in KIRP (Supplementary Figure 3). However, the predictive power based on the seven 7 mutually co-expressed genes was not better than that achieved by combining ACE2 and clinical risk factors. These findings further highlight that ACE2 is closely associated with metabolic homeostasis and is a favorable prognostic biomarker for kidney cancer.

### Correlation between gene expression and drug sensitivity

**Figure 8:**
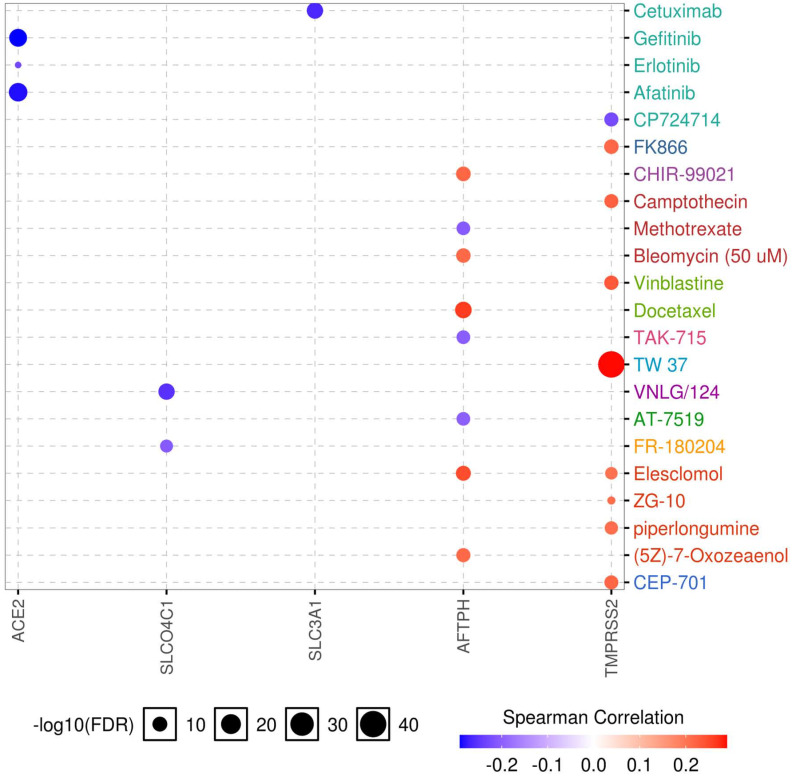
** Drug sensitivity analysis of genes.** The Spearman's coefficient represents the gene expression correlates with the drug. The positive correlation (Red) indicates that the gene's high expression is resistant to the drug.

Drug sensitivity and gene expression profiling data of cancer cell lines in the GDSC database were integrated for an investigation into the correlation between gene expression and drug sensitivity. The expression of each gene in the gene set was evaluated using Spearman's correlation analysis with the drug sensitivity (IC50). A positive correlation means that high gene expression is correlated to resistance to the drug, and vice versa. We found that low expression of *ACE2*, *SLCO4C1*, and *SLC3A1* was associated with drug resistance, whereas high expression of *TMPRSS2* was related to drug resistance. In addition, both high and low expression of *AFTPH* correlated with drug sensitivity (Fig. 8). Considering the role of *SLC3A1* expression in patient survival in KIRP and drug sensitivity, these results revealed a potential therapeutic target for RCC.

### Correlation analysis between ACE2 and immune markers

ACE2 is a predominant RAS member with a significant role in various diseases, such as hypertension, diabetes, and cardiovascular disease 35. Conversely, Ang 1-7, which is responsible for the production of ACE2, exerts anti-inflammatory effects 10, 11. Thus, we investigated the correlation between ACE2 and the markers of different immune cells in KIRC and KIRP. ACE2 was negatively correlated with the hallmarks of immune cells in KIRC and KIRP, including CD79A and CD9 in B cells, PTGS2 in M1 macrophages, BCL6 in follicular helper T cells, and STAT5B and TGFB in regulatory T cells (Table 1). Furthermore, ACE2 was associated with immune infiltration in KIRC and KIRP.

### Altered ACE2 expression after SARS-CoV infection

**Figure 9:**
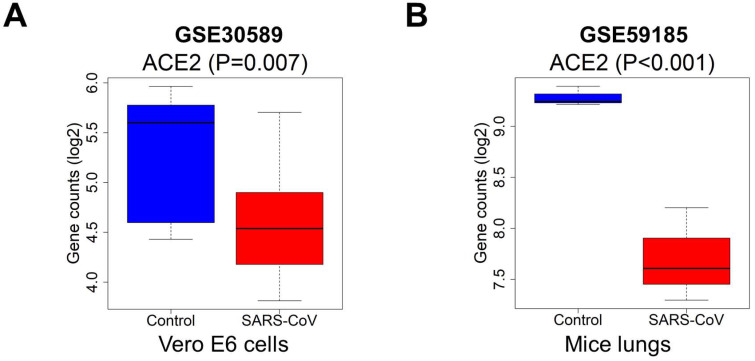
** Expression levels of ACE2 after SARS-CoV infection.** The ACE2 expression was downregulated in (A) Vero E6 cells and (B) mice lung samples with infected SARS-CoV compared to the control, *P*<0.05. Abbreviations: ACE2, angiotensin-converting enzyme 2; SARS-CoV, severe acute respiratory syndrome-coronavirus.

Because of the effect of cancer on SARS-CoV-2 infection, and the significant prognostic role of ACE2 in kidney cancer, we explored the effect of coronavirus on ACE2. We extracted data from the GSE30589 and GSE59185 databases to identify changes in ACE2 expression after infection with SARS-CoV, both *in vitro* and in an animal model. The expression of ACE2 was significantly downregulated in Vero E6 cells and in the lungs of mice infected with SARS-CoV (Fig. 9A-B), indicating that ACE2 expression may also be decreased by SARS-CoV-2, because of the high homology between SARS-CoV and SARS-CoV-2.

## Discussion

SARS-CoV-2 is a newly established beta-coronavirus that is capable of infecting humans 28, where it can rapidly induce acute lung failure and multiorgan damage 20. Similar to SARS-CoV 29, SARS-CoV-2 utilizes ACE2 as a functional receptor and TMPRSS2 for S protein priming to invade host cells 1. Some recent studies have suggested that SARS-CoV-2 could exploit species-specific interferon-driven upregulation of ACE2 to enhance infection 30, and the susceptibility to SARS-CoV-2 in different cohorts seems to correlate with their ACE2 levels 3, 31, 32. Thus, exploring the susceptibility to coronavirus and the physiological functions of ACE2 and TMPRSS2 in humans is essential to the ongoing global drive to manage this pandemic. Kidney cancer patients are at high risk of COVID-19 infection, because the kidney is a target of SARS-CoV-2 infection 7, while cancer is an independent risk factor for COVID-19 infection 19. We applied multi-omics tools to explore the role of ACE2 and TMPRSS2 in KIRC and KIRP and evaluated the impact of COVID-19 infection in kidney cancer patients.

In the present study, we found that ACE2 and TMPRSS2 were distributed widely in human tissues, and their expression levels in the kidneys were higher than those in the lungs, which is consistent with previous studies 5, 6. Interestingly, the mRNA and protein expression of ACE2 was elevated in renal tumor samples, whereas TMPRSS2 expression was significantly downregulated. Moreover, the DNA methylation levels of the ACE2 and TMPRSS2 promoters exhibited marked differential changes in cancer samples, indicating a potential modulation of gene expression in KIRC and KIRP. However, the expression of ACE2 and TMPRSS2 was only weakly negatively correlated. Furthermore, the expression levels of ACE2 and TMPRSS2 in different cancers do not always follow the opposite trend. For instance, these genes are both expressed at low levels in breast invasive carcinoma (BRCA), liver hepatocellular carcinoma (LIHC), and colon adenocarcinoma (COAD) 33. This may be related to the differences between cancers, but the specific reason is not yet clear. We next demonstrated that KIRC and KIRP patient survival was significantly associated with ACE2 expression, and a higher TNM stage corresponded with lower ACE2 expression levels. However, TMPRSS2 was not associated with overall patient survival. These data imply that ACE2 might be a useful early diagnostic marker in patients with KIRC or KIRP. Previous studies have reported the essential role of ACE2 in the treatment of cancer. For example, ACE2 has been identified as an inhibitor of cancer cell growth, metastasis, and angiogenesis in lung cancer via upregulation *of ACE2* mRNA 34. Hepatocellular carcinoma patients with high ACE2 expression levels have a better prognosis than those with lower levels 35. We evaluated the predictive efficacy of ACE2 for patient survival using ROC curves and prognostic nomograms based on the correlation between ACE2 expression and overall survival in KIRC and KIRP. The resulting forecast models could accurately predict survival ability at 3 years after surgery. The AUC value of the integrated nomogram was 0.794 for KIRC with respect to TNM stage, tumor grade, age, and ACE2-based risk score, and 0.802 for KIRP with integrated TNM stage and ACE2-based risk score. These data suggest that ACE2 in combination with clinical characteristics could provide a prognostic indicator of survival rates in KIRC and KIRP patients. We used this model to divide the patient data into low- and high-risk groups based on their scores.

According to the analysis of hallmark and KEGG pathways, the gene sets of butanoate metabolism, glycolysis gluconeogenesis, peroxisome, propanoate metabolism, arginine proline metabolism, bile acid metabolism, fatty acid metabolism, oxidative phosphorylation, adipogenesis, and heme metabolism were significantly enriched in the high-risk group of KIRC and KIRP patients. Furthermore, the mutually co-expressing genes with ACE2, including *AFTPH*, *SLC3A1*, *DAB2*, *LRP2*, *RAB3IP*, *SLCO4C1*, and *CLTRN* were associated with metabolism and amino acid transport, and were also involved in kidney-related diseases 36, 37. According to a literature search, there has been no research on the relationship between these seven genes and SARS-CoV-2. Interestingly, lower expression of these genes was significantly associated with poor survival in the present dataset. This may be associated with the physiological role of ACE2, which is known to affect the absorption of amino acids in the kidneys and intestines 38, 39, and promote mRNA expression of fatty acid oxidation-related genes 40. In diabetic nephropathy patients, urinary ACE2 is associated with metabolic abnormalities related to glucose, triglycerides, and total cholesterol levels 41. About 51% of COVID-19 patients have presented with hyperglycemia 42, and obesity is also recognized as a risk factor for SARS-CoV-2 severity 43. Both SARS-CoV-2 infection and obesity seem to share some common metabolic pathways 44. Furthermore, these genes expression were correlated with drug sensitivity, which reminded us to pay attention to the choice of drugs and the usage and dosage, but whether drug can affect gene expression and SARS-CoV-2 infection we cannot draw conclusions, and more direct evidence may be needed to prove this. The correlation between ACE2 expression and markers of different immune cells in KIRC and KIRP suggests that ACE2 is associated with immune infiltration, and is negatively related to B cells, M1 macrophages, follicular helper T cells, and regulatory T cells. RAS modulates immune functions 45, and ACE2 has been identified as a key regulator of dietary amino acid homeostasis and innate immune and genitourinary tract (GUT) microbiota 46. In summary, decreased ACE2 expression might negatively influence KIRC and KIRP patient prognosis by disrupting the metabolism and the immune system.

To further investigate the post-infection modulation of ACE2 by SARS-CoV-2, we utilized the GSE30589 and GSE59185 datasets to identify the change in ACE2 expression in Vero E6 cells and mouse lungs infected with SARS-CoV. ACE2 expression in the two datasets decreased significantly after SARS-CoV infection. Due to the high homology between SARS-CoV and SARS-CoV-2 47, ACE2 expression levels may similarly be downregulated with SARS-CoV-2 infection. A recent study reported that SARS-CoV-2 interacts with ACE2 and infects ACE2-expressing epithelial and endothelial cells in the lungs and other organs, which in turn downregulates the levels of ACE2 in the endothelium of the lung and other organs, such as the kidneys 48. The downregulation of ACE2 leads to Ang II accumulation, which might accelerate the progression of COVID-19 by increasing the activity of the RAS. In a mouse model, membrane-bound ACE2 exerted an anti-inflammatory role through RAS signaling and the conversion of Ang II to Ang (1-7) 49, which protected the animals against acute lung injury. Therefore, SARS-CoV-2 promotes severe renal cancer progression by downregulating ACE2, weakening ACE2 protection, and disrupting the metabolism and immune system in kidney cancer patients.

In conclusion, we present evidence that patients with KIRC or KIRP might be at high risk of SARS-CoV-2 infection due to the upregulation of ACE2 in tumors. These patients experience metabolic and immune dysregulation after SARS-CoV-2 invasion, causing further downregulation of ACE2. We also determined that the upregulation of ACE2 might serve as a diagnostic biomarker for KIRC and KIRP. However, the present study has some limitations. Coronavirus-related experiments cannot be performed to verify the molecular mechanisms by which ACE2 affects tumorigenesis after SARS-CoV-2 infection due to have no permission to obtain relevant data. In addition, there were no *ACE2* mRNA profiles and KIRC and KIRP clinical information combined with infected SARS-CoV-2 data in the databases used in the present study. Further validation of our conclusions requires clinical information and comprehensive biological experiments.

## Conclusions

In the present study, we determined that ACE2 expression is correlated with TNM stage and overall survival, and could be a potential diagnostic and prognostic biomarker for KIRC and KIRP. SARS-CoV-2-mediated downregulation of ACE2 in KIRC and KIRP patients might exacerbate their illness. Patients with KIRC or KIRP should take adequate preventative measures to avoid COVID-19 infection, as well as being continually monitored for cell metabolism- and immune-related indices.

## Supplementary Material

Supplementary figures and tables.

## Figures and Tables

**Table 1:** Correlation analysis between ACE2 and immune cell markers for KIRC and KIRP

Description	Gene markers	KIRC		KIRP	
		Cor	P	Cor	P
CD8+T cell	CD8A	0.047	0.244	-0.018	0.752
	CD8B	0.065	0.110	-0.029	0.603
T cell (general)	CD3D	0.009	0.824	-0.051	0.364
	CD3E	0.001	0.984	-0.049	0.383
	CD2	0.005	0.900	-0.031	0.580
B cell	CD19	-0.131	**	-0.093	0.096
	CD79A	-0.112	**	-0.123	*
	CD9	-0.168	***	-0.149	**
Monocyte	CD86	0.027	0.506	0.013	0.821
	CD115 (CSF1R)	-0.012	0.776	-0.019	0.733
	TLR4	0.134	***	0.006	0.911
M1 macrophage	INOS (NOS2)	0.029	0.476	-0.126	*
	IRF5	0.075	0.064	0.026	0.638
	COX2 (PTGS2)	-0.115	**	-0.167	**
M2 macrophage	CD163	-0.080	*	-0.003	0.959
	VSIG4	-0.109	**	-0.021	0.709
	MS4A4A	-0.070	0.084	-0.003	0.957
Neutrophils	CD66b (CEACAM8)	-0.022	0.584	-0.076	0.175
	CD11b (ITGAM)	0.010	0.802	-0.012	0.829
	CCR7	-0.080	*	-0.072	0.200
Tfh	BCL6	-0.205	***	-0.165	**
	CD183 (CXCR3)	-0.009	0.828	-0.042	0.452
	CD185 (CXCR5)	-0.088	*	-0.096	0.085
Treg	FOXP3	-0.136	***	-0.055	0.326
	CCR8	-0.046	0.252	0.050	0.371
	STAT5B	0.312	***	0.193	***
	TGFβ (TGFB)	-0.244	***	-0.170	**

Tfh, follicular helper T cell; Treg, regulatory T cell; Cor, R-value of Person's correlation; KIRC, kidney renal clear cell carcinoma; KIRP, kidney renal papillary cell carcinoma. *P<0.05; **P<0.01; ***P<0.001.
