# Supplementary material for: Downregulation of ACE2 expression by SARS-CoV-2 worsens the prognosis of KIRC and KIRP patients via metabolism and immunoregulation
Source: Int J Biol Sci. 2021 May 10;17(8):1925–39. doi: 10.7150/ijbs.57802 (PMC8193256; doi:10.7150/ijbs.57802)
Supplement: Supplementary file 1 — Supplementary figures and tables. [file ijbsv17p1925s1.pdf]

SUPPLEMENTARY TABLES

Supplementary Table 1. Univariate and multivariate analyses of clinicopathological characteristics and ACE2 expression for overall survival in KIRC patients from the TCGA dataset. (*N*=526)

Variables	Total n= 526 (%)	Univariate analysis		Multivariate analysis	
		HR (95% CI)	*P*	HR (95% CI)	*P*
Age					
<60	245 (46.6%)	1 (Reference)		1 (Reference)	
≥60	281 (53.4%)	1.782 (1.299-2.445)	***	1.536(1.112-2.120)	**
Sex					
Female	183 (34.7%)	1(Reference)			
Male	343 (65.2%)	0.960 (0.702-1.314)	0.799		
TNM Stage					
I+II	318 (60.5%)	1 (Reference)		1 (Reference)	
III+IV	205 (38.9%)	3.924 (2.843-5.416)	***	5.409 (2.871-10.192)	***
Missing	3 (0.6%)				
T Stage					
T1+T2	336 (63.9%)	1 (Reference)		1 (Reference)	
T3+T4	190 (36.1%)	3.259 (2.395-4.435)	***	0.495 (0.268-0.914)	*
Tumor grade					
G1+G2	239 (45.4%)	1 (Reference)		1 (Reference)	
G3	279 (53.1%)	2.769 (1.953-3.926)	***	1.773 (1.224-2.567)	**
Missing	8 (1.5%)				
ACE2 Expression					
Low	263 (50%)	1 (Reference)		1 (Reference)	
High	263 (50%)	0.486 (0.355-0.666)	***	0.534 (0.388-0.737)	***

Characteristics with significant *P* values in univariate analysis were screened in multivariate analysis. HR, hazard ratio; CI, confidence interval; TNM, tumor-node-metastasis; KIRC, kidney renal clear cell carcinoma. * *P*<0.05; ** *P*<0.01; *** *P*<0.001.

Supplementary Table 2. Univariate and multivariate analyses of clinicopathological characteristics and ACE2 expression for overall survival in KIRP patients from the TCGA dataset. (*N*=285)

Variables	Total n=285 (%)	Univariate analysis		Multivariate analysis	
		HR (95% CI)	*P*	HR (95% CI)	*P*
Age					
<60	118 (41.4%)	1 (Reference)			
≥60	165 (57.9%)	1.057 (0.571-1.958)	0.860		
Missing	2 (0.7%)				
Sex					
Female	76 (26.7%)	1(Reference)			
Male	209 (73.3%)	0.609 (0.316-1.173)	0.138		
TNM Stage					
I+II	202 (70.9%)	1 (Reference)		1 (Reference)	
III+IV	65 (22.8%)	6.747 (3.503-12.995)	***	6.061 (2.610-14.076)	***
Missing	18 (6.3%)				
T Stage					
T1+T2	233 (81.8%)	1 (Reference)		1 (Reference)	
T3+T4	48 (16.8%)	4.510 (2.399-8.478)	***	1.040 (0.452-2.397)	0.926
Missing	4 (1.4%)				
ACE2 Expression					
Low	142 (49.8%)	1 (Reference)		1 (Reference)	
High	143 (49.2%)	0.516 (0.276-0.964)	*	0.561 (0.286-1.102)	0.094

Characteristics with significant *P* values in univariate analysis were screened in multivariate analysis. HR, hazard ratio; CI, confidence interval; TNM, tumor-node-metastasis; KIRP, kidney renal papillary cell carcinoma. *P<0.05; ** P<0.01; *** P<0.001.

SUPPLEMENTARY FIGURE LEGENDS:

Supplementary Figure 1. The correlation between ACE2 expression and Copy Number Variation (A-B). FDR, false discovery rate.

Supplementary Figure 2. Correlation analysis between ACE2 and TMPRSS2 with clinical characteristics. Sex, Race, Age, and TNM stage were analyzed in KIRC (A-B) and KIRP (C-D), respectively.

Supplementary Figure 3. Survival analysis of 7-mutual co-expressed genes.

Kaplan–Meier survival curves of 7-mutual co-expressed genes with ACE2 in KIRC (A) and KIRP (B) patients, respectively.

A

Gene symbol	Cancer type	Spearman correlation	FDR
ACE2	KIRC	-0.09	0.06
TMPRSS2	KIRC	-0.06	0.23
ACE2	KIRP	0.15	0.02
TMPRSS2	KIRP	0.10	0.16

B

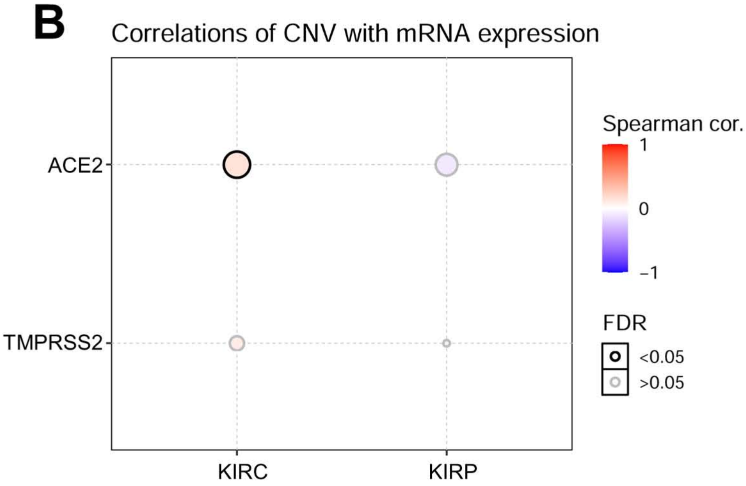

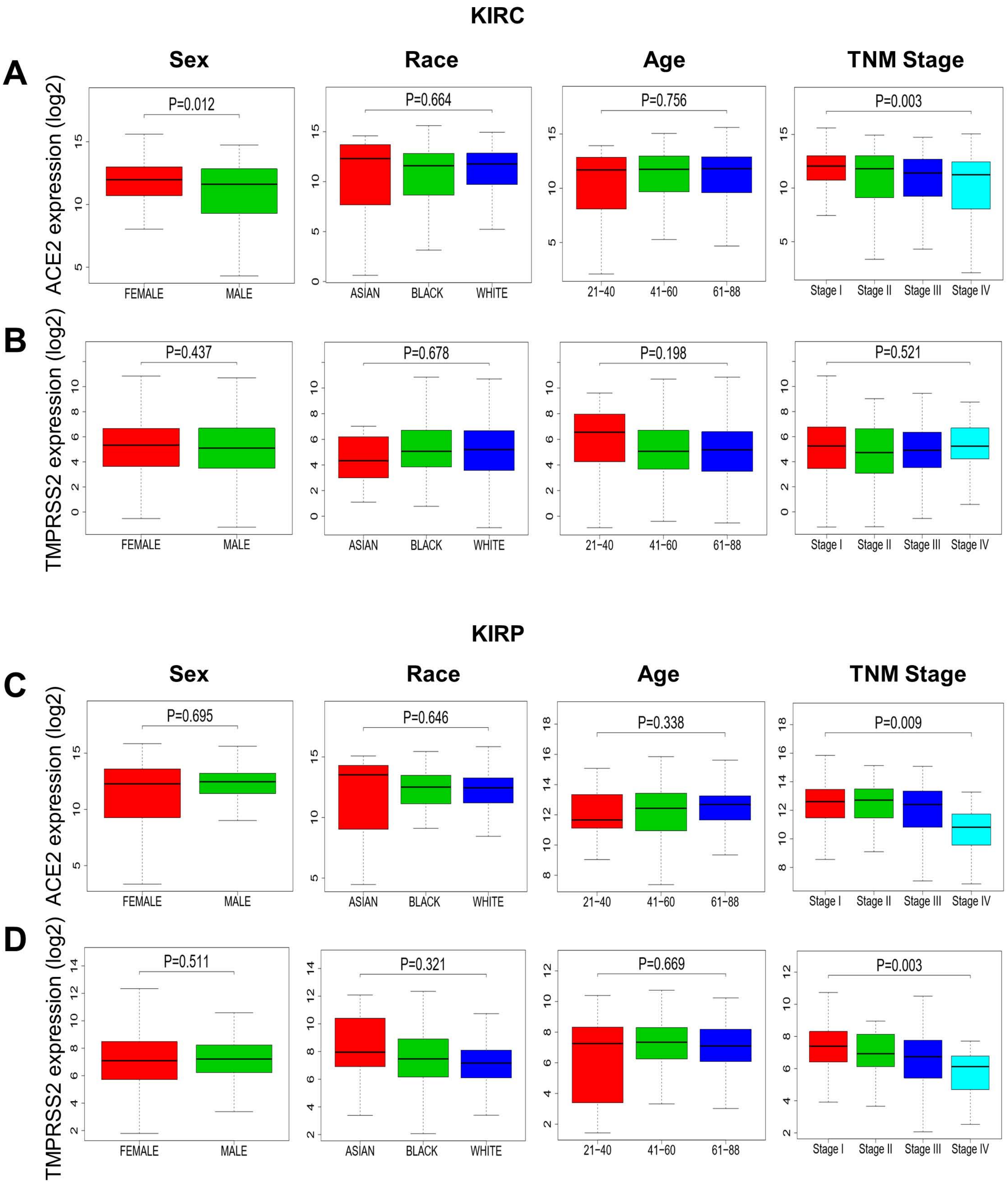
KIRC
A
Sex
Race
Age
TNM Stage
ACE2 expression (log2)
P=0.012
P=0.664
P=0.756
P=0.003
FEMALE
MALE
ASIAN
BLACK
WHITE
21-40
41-60
61-88
Stage I
Stage II
Stage III
Stage IV
B
TMPRSS2 expression (log2)
P=0.437
P=0.678
P=0.198
P=0.521
KIRP
C
Sex
Race
Age
TNM Stage
ACE2 expression (log2)
P=0.695
P=0.646
P=0.338
P=0.009
D
TMPRSS2 expression (log2)
P=0.511
P=0.321
P=0.669
P=0.003

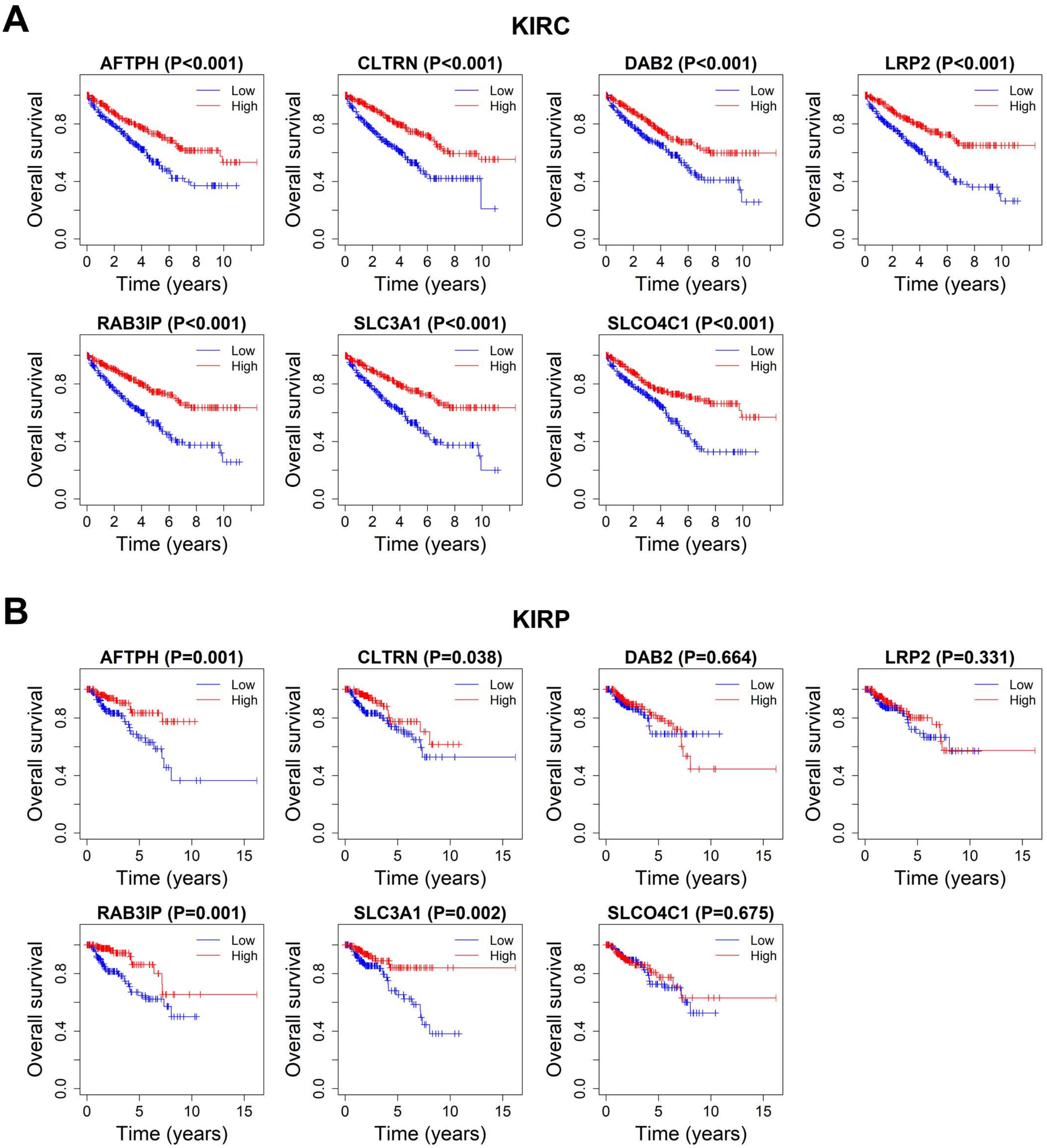
A
KIRC
AFTPH (P<0.001)
CLTRN (P<0.001)
DAB2 (P<0.001)
LRP2 (P<0.001)
RAB3IP (P<0.001)
SLC3A1 (P<0.001)
SLCO4C1 (P<0.001)
B
KIRP
AFTPH (P=0.001)
CLTRN (P=0.038)
DAB2 (P=0.664)
LRP2 (P=0.331)
RAB3IP (P=0.001)
SLC3A1 (P=0.002)
SLCO4C1 (P=0.675)
Low
High
Overall survival
Time (years)
0.0
0.4
0.8
0
2
4
6
8
10
5
15
